# Modeling cynicism and organizational design on job performance: Mediation and moderation mechanism

**DOI:** 10.1016/j.heliyon.2024.e32069

**Published:** 2024-05-29

**Authors:** Abdul Rauf, Hamid Mahmood, Rana Tahir Naveed, Yuen Yee Yen

**Affiliations:** a University Sultan Zainal Abidin, Kuala Terengganu, Malaysia; b TIMES Institute, Multan, Pakistan; c University of Education, Lahore, Pakistan; d Multimedia University, Melaka, Malaysia

**Keywords:** Organizational design, Cynicism, Knowledge hiding, Justice, Servant leadership

## Abstract

Modern organizations assert that cynicism and organizational design provide advantages for knowledge-intensive settings. However, organizational crises may lead to resource shortages, prompting increased knowledge hiding (KH) among workers for competitive edge. Therefore, current study aims to examine the influence of organizational design and cynicism on job performance with organizational justice and KH through the moderating effect of servant leadership. Convenience sampling technique was used for data collection from 730 manufacturing organization employees via a survey questionnaire and data were analyzed with AMOS (28.0). Findings showed that KH's behavior negatively influenced by organizational design and positively influenced by cynicism. The current study also validates that higher management needs to practice advanced organizational justice to improve performance that drastically generates justice practices and reduces KH within the firms. Moreover, deploying servant leadership helps to control the cynicism, and employees start practicing knowledge-sharing behavior that significantly contributes to the performance.

## 1 Introduction

Contemporary situational hardships have the potential to encourage enduring cynicism among employees while organizational design geared towards knowledge sharing behavior [1,2]. Whereas employees attempt to preserve their competitive advantages even dearth of resources and engage in knowledge hiding behavior that consequently causes low job engagement [3,4]. However, numerous organizations repeatedly exercise momentous exertions to assure knowledge-sharing behavior among employees, but their perceptional rigidity never changes that ultimately resulted in KH [5]. Therefore, it becomes imperative for an organization specifically developing countries like Pakistan to prevent the KH and mitigate its adverse consequences. Additionally, servant leadership plays a remedial role in organizations to tackle the complications and challenges to gain significant consideration when employees' responses are concerned [6]. Such leadership style optimistically triggers the behavior of knowledge sharing that consequently mitigate the behavior of KH [7] and paves the way for the development of an organization. Servant leadership encourages employees, inspires them to innovate, adopt the challenges and changing behavior that enhances their workplace performance and preserves the practice of KH to alleviate the negative consequences on their job performance. The dilemma of KH denotes destruction and deliberate knowledge concealment among peers, negatively impacting performance and the culture of an organization that consequently damages their association and is the cause of cynicism [8].

Although existing literature provide important insights on KH, however, the elements that mitigate KH are rarely addressed [8,9], which define intentional attempts to decrease job performance. These factors have significant importance in organization success as they have deliberate efforts to decrease job performance. Furthermore, there is lack of comprehensive empirical and theoretical understanding of how manager can effectively tackle knowledge hiding behavior and improve job performance. Henceforth, the present research on KH proposes the collective viewpoint of transformational leadership theory [10], and social exchange theory [11] to apprehend the effect of KH for genuine consideration of employees' intentions. Present study focused on nuanced management approaches that decisive for nurturing employee performance and organizational success in contemporary strategic management deliberations in manufacturing organizations in Pakistan. As per the researcher's knowledge, no single research used (TLT) and (SET) theories combined in the Pakistani context that explained the behavior of KH. Additionally, present research examines the organizational design construct that interrelated with job performance for alleviating the KH, and servant leadership with employee performance. The theme of this research is consistent with extant research through the relationship between organizational design, leadership, and KH [12].

**Fig. 1:**
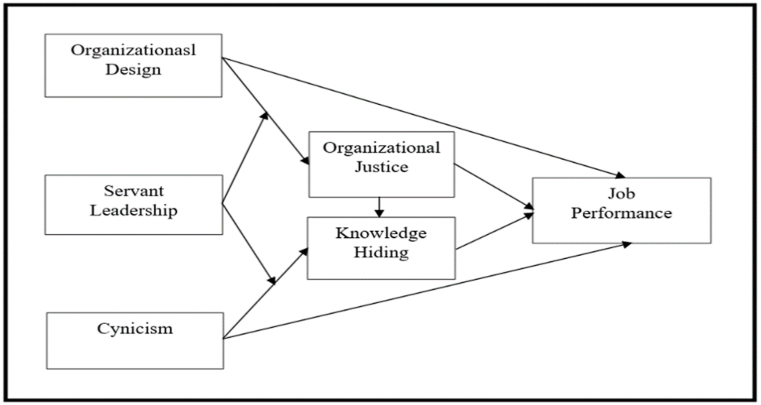
Research framework.

This study extensively offering detailed examination of how the organizational design and justice mitigate the cynicism and KH practices through servant leadership that ultimately trigger the employee performance. The objectives of the present research are; 1) to identify the employee's insight into organizational design and cynicism toward activating job performance among manufacturing industries in Pakistan, 2) to examine the mediating effect of organizational design and KH in the relationship among organizational design and cynicism, and job performance, and 3) investigate the moderation role of servant leadership on the association among organizational design and organizational justice, and cynicism and KH respectively (Fig. 1). This study offers an innovative injector via up-to-date data and contemporary context that incorporate the effect of economic crises and the behavior of KH by addressing the present study objectives. Thus, the present research contributes to the body of KH literature by investigating its experiences and consequences that disturb Pakistani manufacturing organizational performance. Findings revealed a valuable inference for potential researchers via investigating the handling of economic crises, dearth of resources and servant leadership [2], and discourse KH behavior [13] among overall organizations to control the human resources to prevent the decreasing job performance among employees [14,15].

## 2 Literature review

### 2.1 Underpinning theories

The transference of implicit knowledge is complicated as compared to explicit knowledge due to its hiding impossibility [16], and more than 90 % of knowledge among organizations is tacitly affixed in the employees' subconscious mind [17]. While focusing on the contextual nature of KH, the present research logically introduces the theoretical model of transformational leadership theory [10] to advance the healthy intuitions into the behavior of KH. TLT trigger the performance and mitigate cynicism by nurturing an optimistic organizational culture. Regarding TLT, in the scenario of economic and organizational crises, employees' reaction regarding the loss of resources relies on transformational leadership. Le and Lei [7], argued that TL is triggered by encouraging employees to alleviate the KH practice and promote knowledge sharing. Social interaction was envisioned via social exchange theory [18,19] with the foremost value of convinced behaviors that enhanced the optimistic desired experience and lessened the adverse practices over social exchanges [20] and consequently, associations were figured and sustained among employees if relationship reward exceeds the associated complexities [21]. SET elucidates the transparent organizational structures for performance advancement and highlighted alleged discriminations that be the cause of cynicism that adversely affect the performance. The belief of social trade identifies the frequencies of negative and as well as positive exchanges via inferring the extant recommendation “positive handling for positive handling” and consequently denoting employees' “inclination to reoccurrence negative handling for negative handling” [19,22]. Both TLT and SET indicated leadership efficacy and organizational mechanism holistically encourage the performance and attitudes of employees by dropping the practice of cynicism and boosting workplace exchanges.

### 2.2 Variables discussion

#### 2.2.1 Organizational design and job performance

Organizational design defines as a specific people arrangement, technology, dividing work tasks, and mutual learning to achieve internal compliance and provide knowledge exchange opportunities to coordinate with employees [23,24]. Therefore, it regulates the responsibility distribution, authority lines, way to connect activities, internal environment, external reality, context, behavior, strategy, and structure. Additionally, organizational design terms are associated with other terms which used identically [25], such as architecture [26] or organizational structure [27]. Numerous studies in recent years state that various organizations are still based on the Tayloristic principle [28], in which organizational design is established on a high level of structure and labor division [29]. The organizational design reflects the valuable choice of the organization, which refers to how job tasks are divided, grouped, and coordinated and how perceived job characteristics [30] that be the cause of better performance. Likewise, Baporikar and Randa [31] highlighted that there is an optimistic association exist among organizational design and job performance. Considerable research in recent decades established the impact of organizational design on job performance [32]. So, we hypothesized that:

H1 Organizational design positively and significantly impact on the job performance.

#### 2.2.2 Cynicism and job performance

Cynicism is an attitude that refers to mistrust, disappointment, and negative feelings regarding a particular object [33,34], and it is an intrinsic individual behavior that reflects frustration and negative emotions regarding human behavior. Additionally, cynicism considers a multilateral phenomenon (1. Negative attitude, (2. lack of integrity, and (3. disapproving behavioral tendency [35]. Moreover, employee cynicism is a skeptical attitude of the employee resulting from actions and critical motives [36], and coworkers have negative behavior toward them, they develop cynicism and respond with negative attitude toward their coworkers and their organization [37]. For instance, excessive stress, absenteeism, and overload frustration are identified at the individual level [38], which leads to poor job performance. As the time passes, the practice of cynicism developed as the novel pattern of employee and organizational management association. It can be found that a sound percentage of personnel were exceedingly cynical about their organizations because the practice of cynicism ascends the dearth of confidence in his/her organization and feel distrusted. By doing so, the performance of employees affected that ultimately put negative impact on overall organizational performance. Furthermore, an employee's positive role is very important for the success of any organization [39]. Managing employee cynicism in an organizational context is crucial because it could hinder performance [40]. An extant study revealed that cynicism adversely impact on the employee performance of manufacturing companies of India [41]. Thus, we hypothesized that:

H2 Cynicism negatively impact the job performance.

#### 2.2.3 Organizational design and organizational justice

The organizational design agenda incorporates insight and theories from management and organizational research [42]. Edifice's early academic work on organizational design and structure was built in the 1960s and 1970s; a study on organization design found renewed interest among organization and management researchers [43]. Subsequently, organizational justice drives an organization whether it is authentic; it builds theoretical sight that good organizational design could lead to positive organizational justice [24,44]. Furthermore, organizational justice is influenced by the experiences and treatment of the organization in various areas, which show the organization's impartiality. Adequate organizational design integrates the group's work, and a person feels part of the work unit [45]. Integration is essential to reduce conflict between employees and increase organizational justice perception [46]. Past study examines organizational design with the perception of organizational justice i.e., Lambert et al. [44] stated that organizational design positively influences organizational justice. So, we hypothesized that:

H3 Organizational design positively and significantly impacts on organizational justice.

#### 2.2.4 Cynicism and knowledge hiding

Employee reactions toward organizational changes got much attention from researchers in the last decades [47,48], and employee support and a positive attitude are dynamic for every organization [13]. Employee cynicism is very important to manage because it promotes conflicts for organizational changes [49]. Mostly, employees become cynics because of stress at the workplace, which may lead to hiding knowledge [50,51] and more likely to hide knowledge because it can help them to protect and maintain competitive advantages [49]. Additionally, cynicism incorporates negative behavior in the organization regarding employee positions [52]. Consequently, workers need to be more motivated to cooperate with coworkers and demonstrate hesitation to share expertise [53]. Furthermore, employees may build cynical attitudes in an organizational crisis and be more inclined to adopt knowledge hiding [54]. Additionally, cynic employees are more intent on nonparticipation from learning about hidden talent and less attracted to coworkers and knowledge [55]. A study was conducted in software houses of China that highlighted that cynicism significantly impact on knowledge hiding [56]. Thus, we hypothesized that:

H4 Cynicism positively and significantly impacts on knowledge hiding.

#### 2.2.5 Organizational justice and job performance

Justice refers to an action and decision understood morally based on religion, equity, ethics, law, fairness [57], and basic concern areas for employees and organizations [58]. Organizational justice defines as employee perception regarding fairness in the organization [59]. Input denotes to their effort, time, rewards, recognition, promotion pay, and resource, which assist employees in job performance [60]. Furthermore, organizational justice is also linked with social exchange theory, which rises social life as a development of progressive transactions among two or more parties [11]. When employees observe that the organization fairly treats them, they feel a sense of obligation and increase their efforts [61], because if employees treat fairly, they are passionate about their work and exhibit higher performance. Numerous studies suggest organizational justice affects employee job performance [62,63]. Furthermore, a high organizational justice perception enhances employees' positive attitudes and increases job performance [64]. So, we hypothesized that:

H5 Organizational justice positively and significantly impacts on job performance.

#### 2.2.6 Knowledge hiding and job performance

Job performance denotes employee behavior contributing to the organization's effectiveness [65], and employee performance could be influenced by knowledge hiding for three reasons [66]. First, knowledge hiding could reduce knowledge availability, and facilitating better performance. Secondly, an employee who performs knowledge hiding tends to have a negative mindset, a violent cycle in which the employee is not prone to support others or search for support; consequently, they do not have the confidence to support their colleague's offer [12]. Thirdly, knowledge hiding practices are generally underestimates. Their coworkers can determine knowledge hiding when performed by colleagues [2]. Furthermore, Anand et al. [67] stated that knowledge hiding is a systematic process to distribute, disseminate, and transfer knowledge in a multidimensional context to an organization or person in need. KH can pointedly obstruct the performance of employees by impeding information stream, deterring association, stifling novelty, and corroding belief inside the organization. This disinclination to share acquaintance not only weakens employee efficiency but also demoralizes team efficacy and eventually distresses organizational consequences. Therefore, knowledge hiding decreases job performance due to decreased decision-making, creativity, and problem-solving [68]. Additionally, knowledge hiding negatively impacts hider creativity, triggers a distrust loop, and hurts the interactive relationship between knowledge hider and seeker [69]. Moreover, knowledge hiding directly affects job performance [2,68]. Thus, knowledge hiding as a negative interactive experience limits the employee's inclination to participate in work behavior actively [69]. Thus, we hypothesized that:

H6 Knowledge hiding negatively impacts on job performance.

#### 2.2.7 Organizational justice as mediator

Organizational justice has many benefits for organizations, employees, and society [70], especially in developing countries, where many political, economic, and social challenges prevail, and injustice accelerates unsuccessful events in the workplace. Shan et al. [71], discussed organizational justice as the least underutilized and understood concept to create organizational effectiveness in developing countries and explore organizations' soft side. Organizational justices with three dimensions of interactional justice, distributive justice, and procedural justice are exciting research areas with novel input to the theory and practices [24,64]. Distribution justice refers to a fair distribution of outcomes and resources; procedural justice is about fair use in decision-making; and interactional justice focuses on communication procedures and outcomes [72]. It is authoritative to consider here that organizational justice is not about how it should be, but rather about how individuals, particularly employees, perceive to be rewarded and treated by authorities, sponsors, and managers [72]. Furthermore, past studies also examine organizational design with organizational justice [44,73] that ultimately increases job performance [74]. So, we hypothesized that:

H7 Organizational justice positively and significantly mediates the relationship between organizational design and the job performance.

#### 2.2.8 Knowledge hiding as mediator

Knowledge hiding is a thoughtful effort by the employee to overwhelm or hide important information which coworkers ask for [13]. Firstly, justifying hiding, in which a variety of descriptive argument or justifications is offered to validate the incapability of knowledge sources [75]. Secondly, in deceptive hiding, individuals less disclose than others are required. Thirdly, in playing dumb, individuals pretend to be oblivious to information [75]. Past researchers identified an embarrassment of interpersonal and situational factors which contribute to knowledge hiding [9]. Particularly psychological ownership, time pressure, low knowledge-sharing climate, workplace ostracism, interpersonal distrust, and knowledge complexity are associated with knowledge hiding [76,77]. Past study similarly illustrates the mediating role of knowledge hiding in the relationship between abusive supervision [68], and organization politics [78] with employee creativity. We encompass such research by proposing that cynicism inclines negative organizational behavior and reduces job performance as it discourages an employee from sharing knowledge with colleagues. Thus, we hypothesized that:

H8 Knowledge hiding negatively mediate the relationship between cynicism and the job performance.

#### 2.2.9 Servant leadership as moderator

Servant leadership is presently recognized as a contemporary pattern within leadership studies, characterized by an emphasis on leader behavior and a focus on followers' concerns, responsiveness, and development [79,80]. It also has a significant aspect of the capacity to trigger the performance of an organization, specifically in manufacturing and service organizations [50,81]. Thus, it is the need of an hour for organizational management to encourage the employees toward performance by agreement the organizational justice at the workplace [24,80]. The theory of transformational leadership sets the grounds for leadership that offers a logical consideration aspect that may moderate the effect of the concerned organizational mechanisms. Eva et al. [82] argued that a leader's role as a servant leader indicates a helper or factor that accompanied by different spiritual, relational, emotional, and ethical extents. Extant research also highlighted that this leadership style perfectly moderates the relationship between employee cynicism and knowledge hiding [2]. Thus, the theory of transformational leadership is adequately applied to the organizational and individual levels, that cause robust enhancement of organizational and individual level performance [83]. So, we hypothesized that:

H9a Servant leadership positively and significantly moderate the relationship of organizational design and the organizational justice; like that if servant leadership is high than the association among organizational design and the organizational justice would be weakened.

H9b Servant leadership positively and significantly moderate the relationship of cynicism and the knowledge hiding; like that if servant leadership is high than the association among cynicism and the knowledge hiding would be weakened.

## 3 Research methods

### 3.1 Methods

This research received an ethical approval from the Office of Research, Innovation and Commercialization (ORIC) with reference no TI/ORIC/2023/73. The questionnaire of the present research contained three segments in which brief explanations regarding the study was described in the first segment. The second segment contained the demographic characteristics of respondents (employees). A set of thirty-six close-ended queries about key latent constructs like organizational design, cynicism, organizational justice, KH, servant leadership, and job performance of manufacturing industries in Pakistan was arranged in the third segment of the instrument.

### 3.2 Instrument

This study adapted nine items i.e., ‘job design with high levels of autonomy’ from Avey et al. [84], and Zerella et al. [85] to measure organizational design, five items i.e., ‘I have become more cynical about whether my work contributes anything’ scale adapted from Maslach et al. [86] to measure employee cynicism, six items i.e., ‘job decisions are made by the general manager in an unbiased manner’ adapted form Abubakar et al. [33] to measure organizational justice, six items i.e., ‘promise that you would help but trying to delayed as possible’ scale adapted from Connelly et al. [13] to measure KH, adapt five items i.e., ‘my department manager works hard at finding ways to help others be the best they can be’ scale from Ehrhart [87] to measure servant leadership, and adapt also five items i.e., ‘my performance was still good as the time before’ scale from Chiang and Hsieh [88] to measure job performance. All thirty-six items were secured on a seven-point Likert scale ranging from 1 to 7 (strongly disagree = 1 to strongly agree = 7).

### 3.3 Sampling and data collection

The population was the employees of the manufacturing organizations in Pakistan who currently work there. To consider the present research settings and the concerned constructs directly linked with those manufacturing organizations where employees were based on their peers’ support and information to trigger their performance [89]. Additionally, we choose employees as participants due to trust, conversations, and, most importantly, competition is the basic issues that trigger the practice of KH among peers [90]. A survey questionnaire was used to collect data from employees of manufacturing organizations. The non-probability sampling technique of convenience sampling was used in present research. Researcher prefer convenience sampling due to its easy contact to respondents and economical [91]. One more reason for using convenience it saves the resources and time specifically, when interest of population is not easily accessible. This sampling method is suitable when the survey questions are appropriate to the respondents (employees of manufacturing industries in Pakistan) that encompass a large population, and there is no need for constructs manipulation [92]. The time frame of data collection lies in-between November to December 2023. In the data collection process, four enumerators assisted the researcher in the survey. Approximately 25 min were given to the respondents to complete the survey; the participation is purely based on voluntary and thanked with chocolates. Out of 850 distributed questionnaires, 803 were returned, and after the screening, 73 questionnaires were discarded due to double filling and missing values. Finally, 730 questionnaires were finalized for statistical evaluation that had an 86 % response rate.

## 4 Data analysis

### 4.1 Demographics of respondents

Table 1 highlights the demographics of respondents. In the gender section, males were dominant with a percentage of 66.8 % with the 488 frequencies. The second sub-section of age, 26–33, has a prominent frequency of 233 with a percentage of 31.9 %. In qualification section of respondents, the leading degree is a bachelor's with a frequency of 244 with a percentage of 33.4 %. The second last section refers to the income of respondents, in which the 44001 and above have the higher frequency of 221 with the percentage of 30.3 %. The last section is about the experience of the respondents, in which 7–9 years group have the leading frequency of 294 with a percentage of 40.3 %.

**Table 1:** Demographics of respondents (n = 730).

No.	Demographics	Frequency	Percentage
**1**	**Gender**		
	Male	488	66.8 %
	Female	242	33.2 %
**2**	**Age**		
	18–25 Years	60	8.2 %
	26–33	233	31.9 %
	34–41	193	26.4 %
	42–49	166	22.7 %
	50 or above	78	10.8 %
**3**	**Qualification**		
	Intermediate	204	27.9 %
	Bachelor	244	33.4 %
	Master	194	26.6 %
	Mphil	88	12.1 %
**4**	**Income**		
	15**,**000–24000 PKR	125	17.1 %
	24,001–34000 PKR	215	29.4 %
	34,001–44000 PKR	169	23.2 %
	44,001 or above	221	30.3 %
**5**	**Experience**		
	1–3 Years	102	13.9 %
	4–6 Years	113	15.5 %
	7–9 Years	294	40.3 %
	10 and above	221	30.3 %

### 4.2 Measurement model with fit indices

Confirmatory factor analysis (CFA) was used to measure the constructs' reliability and validity in Table 2. The results revealed that the assessment of measurement model is in acceptable range of the model fit (χ 2 = 347.21; df = 147 χ2/df = 2.361; SRMR = 0.038; GFI = 0.933; AGFI = 0.844; NFI = 0.922; RFI = 0.931; CFI = 0.947; TLI = 0.929; RMSEA = 0.058) [93]. All items' standardized factor loadings exceeded the threshold level of 0.50, with a significance of p < 0.001. The average variance extracted also met the threshold level of 0.60; in the present research, it falls within the range of 0.727–0.818. The composite reliability also surpassed the threshold level of 0.70 [94], and results revealed that it also falls within the range of 0.934–0.957. The value of Cronbach's alpha (α) coefficients also met the threshold level of 0.70, and results highlighted that it also falls within the range of 0.83–0.93. Finally, the convergence validity of the present research was also supporting the measures [95]. The present research results fulfilled the requirements and established the discriminant validity highlighted in Table 3.

**Table 2:** Confirmatory factor analysis (n = 730).

Construct	Indicators	SFL
Organizational design	OD1	0.93
α = 0.88, CR = 0.955, AVE = 0.727	OD2	0.86
	OD3	0.78
	OD4	0.91
	OD6	0.83
	OD7	0.85
	OD8	0.84
	OD9	0.81
Cynicism	CYN1	0.88
α = 0.87, CR = 0.934, AVE = 0.779	CYN2	0.91
	CYN3	0.86
	CYN4	0.88
Organizational Justice	OJ1	0.92
α = 0.93, CR = 0.952, AVE = 0.768	OJ2	0.91
	OJ3	0.88
	OJ4	0.89
	OJ5	0.87
	OJ6	0.78
Knowledge Hiding	KH1	0.82
α = 0.83, CR = 0.943, AVE = 0.735	KH2	0.87
	KH3	0.85
	KH4	0.92
	KH5	0.81
	KH6	0.87
Servant Leadership	SL1	0.91
α = 0.91, CR = 0.957, AVE = 0.818	SL2	0.94
	SL3	0.89
	SL4	0.91
	SL5	0.87
Job Performance	JP1	0.86
α = 0.86, CR = 0.952, AVE = 0.800	JP2	0.87
	JP3	0.94
	JP4	0.89
	JP5	0.91

Note: AVE = average variance extracted, SFL = standardized factor loadings.

CR = composite reliability, α = Cronbach's alpha, ***p < 0.001.

**Table 3:** Confirmation of the discriminant validity.

Constructs	OD	CYN	SL	OJ	KH	JP
1	**0.852**	−0.234	0.349	0.218	−0.139	0.121
2		**0.882**	0.298	−0.211	0.118	−0.101
3			**0.876**	0.342	−0.198	0.244
4				**0.857**	−0.321	0.221
5					**0.904**	−0.141
6						**0.894**
**Mean**	3.85	3.79	3.64	3.89	3.62	3.71
**SD**	1.05	1.01	1.12	1.09	1.07	1.06
**VIF**	1.114	1.912	1.543	1.3321	1.191	1.071

Note (s): The diagonal row contained italic number which are square root of AVE.

### 4.3 Common method variance

The Herman single factor was used to measure each variable's common method variance [96]. The common method was used for data collection, so the variance counterfeit was evaluated that can be shared among the constructs. The exploratory factor analysis of all the items of constructs revealed that the principal three factors avariciously enlightened for 62.71 % of the variance among constructs, with prime factor rating for 38.34 % and the succeeding factor elucidation for 24.37 % of the total variance. Hence, the first-factor variance is below the threshold level of 50 %. Consequently, this data is pure from common method variance.

### 4.4 Structural model validation

**Fig. 2:**
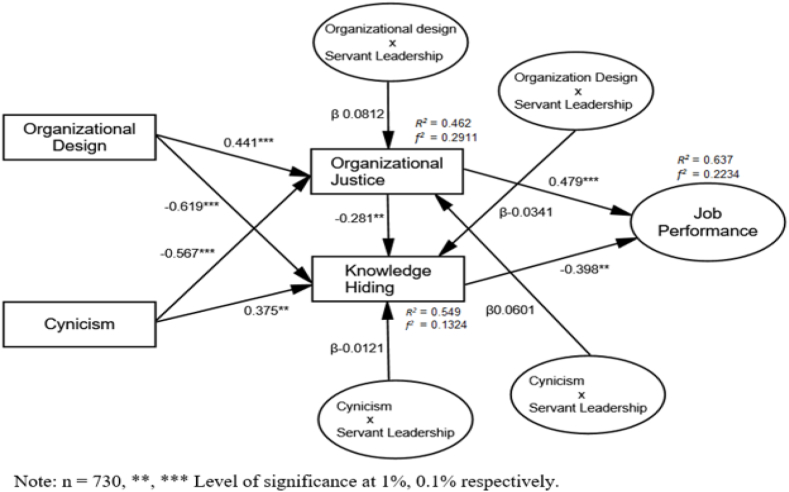
Research model validation.

Structural Equation Modeling (SEM) was used to test the hypothesis of present research through AMOS (version 24.0) [97]. Categorically, the technique of bootstrapping was applied with 95 % of the analysis of confidence interval to identify the effect of organizational design on the first mediator of organizational justice and the cynicism on the second mediator KH and both mediators in the association among organizational design and cynicism and job performance. Correspondingly, servant leadership as a moderator examines the impact on both the connotation of organizational design, justice, cynicism, and KH. Table 4 highlights the results of a hypothesis test. Results of present model highlighted that indices of goodness fit: (χ2 (148) = 342.91, χ2/df = 2.316, p = 0.000, AGFI = 0.921; GFI = 0.936; CFI = 0.954, IFI = 0.954, RFI = 0.934, NFI = 0.936, RMSEA = 0.062, PCFI = 0.819, PGFI = 0.729 and PNFI = 0.821. The value of ChiSq/df and goodness of fit indices revealed the satisfactory fit with the model that relied on the large sample size. The model perfectly captures the 64 % of job performance through exogenous constructs (organizational design, cynicism, organizational justice, KH, and servant leadership) (see Fig. 2).

**Table 4:** Results of direct effects.

Paths	Path Coefficients	t-statistics	Bias-corrected CI 0.95 %	Relationships
H1**:** OJ ← OD	0.441**	2.567	[0.206, 0.871]	Support
H2**:** KH ← OD	−0.619***	−3.567	[-0.119, −0.723]	Support
H3**:** OJ ← CYN	−0.567***	−5.412	[-0.211, −0.711]	Support
H4**:** KH ← CYN	0.375**	2.559	[0.101, 0.691]	Support
H5**:** KH ← OJ	−0.281*	−2.987	[-0.112, −0.542]	Support
H6**:** JP ← OJ	0.479**	2.667	[0.201, 0.712]	Support
H7**:** JP ← KH	−0.398**	−2.661	[-0.107, −0.745]	Support

### 4.5 Results of hypothesis

The results revealed that organizational design was positively linked with organizational justice (H1, OD → OJ = 0.441**, t 2.567, CI; 0.206, 0.871, p = 0.0000 and organizational design was negatively related to KH (H2, OD → KH = −0.619**, t −3.567, CI; −0.119, −0.723, p = 0.000). We also found support that cynicism was negatively related to organizational design (H3, OD → 10.13039/100014294KH = −0.567**, t −5.412, CI; −0.211, −0.711, p = 0.000) and cynicism was negatively linked with 10.13039/100014294KH (H4, OD → 10.13039/100014294**KH** = 0.375**, t 2.559, CI; 0.101, 0.691, p = 0.000). Moreover, organizational justice was negatively linked with KH (H5, OD → KH = −0.281**, t −2.987, CI-0.112, −0.542, p = 0.000). Furthermore, Organizational justice is positively related to job performance (H6, OD → KH = 0.479**, t 2.667, CI, 0.201, 0.712, p = 0.000). Lastly, knowledge hiding is negatively linked with job performance (H7, OD → KH = −0.398**, t, −2.661, CI, −0.107, −0.745, p = 0.000). (see Fig. 2)

### 4.6 Moderation results

Table 5 showed that servant leadership positively moderate the relationship between organizational design and organizational justice and bootstrapped CI indicated that the results were positive and significant (β SL x OD → OJ = 0.0812, t = 4.345, [CI: 0.0278, 0.0872], p, <0.001) thus supported H9a1. Moreover, servant leadership negatively moderate the relationship between organizational design and 10.13039/100014294KH and bootstrapped CI indicated that the results were negatively and significant (β 10.13039/100014294KH x CYN → JP = −0.0341, t = −6.119, [CI: 0.0211, 0.0601], p, <0.001) thus supported H9a2. Servant leadership negatively moderate the relationship between cynicism and organizational justice and bootstrapped CI indicated that the results were negatively and significant (β 10.13039/100014294KH x OJ → JP = −0.0121, t = −5.991, [CI: 0.0112, 0.0473] p, <0.001) thus supported H9b1. Lastly, servant leadership positively moderate the relationship between cynicism and 10.13039/100014294KH and bootstrapped CI indicated that the results were positively and significant (β SL x CYN → 10.13039/100014294KH = 0.0601, t = 4.211, [CI: 0.0201, 0.0701] p, <0.001) thus supported H9b2. (see Fig. 2).

**Table 5:** Results of moderation analysis.

Paths	Path Coefficients	Boot SE	t-statistics	Bias-corrected CI 0.95 %	Relationships
H9a1: SL x OD → OJ	0.0812***	0.0171	4.345	[CI: 0.0278, 0.0872]	Supported
H9a2: SL x OD → KH	−0.0341***	−0.0187	−6.119	[CI: −0.0211, −0.0601]	Supported
H9b1: SL x CYN → OJ	−0.0121***	−0.0184	−5.991	[CI: −0.0112, −0.0473]	Supported
H9b2: SL x CYN → KH	0.0601***	0.0129	4.211	[CI: 0.0201, 0.0701]	Supported

We further evaluated the simple effects to determine the nature of each interaction between constructs. By applying the suggestion of Edwards and Lambert [98], we divided servant leadership into high and low dummy variables. We investigate the organizational design of organizational justice at high and low levels of servant leadership. The organizational design had a higher and significantly affected organizational justice when having a higher level of servant leadership (β (high) SL x OD à OJ = 0.0910, t = 2.981, [CI: 0.0411, 0.0572], p, 0.001). A higher level of servant leadership improves the organizational design, increasing organizational justice. Secondly, we investigate the organizational design of KH at high and low levels of servant leadership. The organizational design had a higher negative significantly affected on KH when having a higher level of servant leadership (β (high) SL x OD à OJ = −0.0321, t = −7.181, [CI: 0.0311, 0.0502], p, 0.001). A higher level of servant leadership improves the organizational design that decreases the level of KH behavior of employees.

Thirdly, we investigate the cynicism on organizational justice at high and low level of servant leadership. Cynicism had a higher negative significantly affected on organizational justice when having a higher level of servant leadership (β (high) SL x OD à OJ = −0.0278, t = −5.229, [CI: 0.0291, 0.0498], p, 0.001). A higher level of servant leadership decreases the cynicism that eventually increases the level of organizational justice within the organization. Fourthly, we investigate the cynicism of KH at high and low level of servant leadership. Cynicism had a higher positive significantly affected on KH when having a higher level of servant leadership (β (high) SL x OD à OJ = −0.0321, t = −7.181, [CI: 0.0311, 0.0502], p, 0.001). When having a higher level of servant leadership within the organization, will decrease the level of cynicism, which also decreases the KH behavior among employees.

## 5 Discussion and implications

### 5.1 Major findings

This research offers a momentous theoretical insight regarding job success. Present study used empirical observation to examine how cynicism is actively used in an organization's design. Results primarily showed a positive effect of organizational design and a negative impact of cynicism on job performance, supporting previous research findings in the organizational settings [53,99]. Accordingly, KH has a negative effect on employees' job performance as well as on the organization [100]. On the other hand, organizational justice advocated for a positive effect on employees' job performance that would eventually improve an organization's performance [101]. The findings of present study are consistent with extant study's findings on call center employees that robustly effected via cynicism and knowledge hiding practices [102], in manufacturing industries of Kuala Terengganu, Malaysia under the umbrella of psychological ownership knowledge theory and social exchange theory [24] and health sector, insurance, and banking organization under the shadow of job-demands resources theory in Dhofar Governorate, Oman [103]. Organizational justice and KH have an unstable relationship with the opposite effect. Results revealed that cynicism and KH, as well as links between organizational design and organizational justice, could be improved and strengthened by servant leadership [104]. Therefore, to improve employees' ability to execute their jobs, servant leadership plays a motivating role in discouraging the practice of KH and encouraging the practice of organizational justice. Even though organizational justice had a substantial influence on KH behavior, it is still possible that the justice-related phenomenon varies depending on this study.

The results of the current study showed that organizational structure and cynicism had opposite effect on employees' success at work, proved via H1 and H2, respectively. Additionally, it was noted that organizational design (H3) played a positive role in organizational justice and cynicism can also lead to KH (H4). Additionally, two contrasting viewpoints showed that organizational justice is rendered towards job success in a positive and negative sense of KH, which refers to H5 and H6, respectively. Finally, considering the intervening constructs of organizational justice and KH also placed a variety of contributions—both favorably and negatively—between cynicism and job performance by indicating H7 and H8 in between organizational design and job performance. Two alternatives were also considered when answering the moderating hypothesis: organizational design and organizational justice, and cynicism and KH favorably affected by servant leadership. Accordingly, findings revealed that organizational design, justice, and cynicism have beneficial effects on employee job performance, while KH and cynicism have negative effects. However, these effects can be changed with the help of servant leadership, which is a powerful influence.

Organizational justice involves in recreation with a bold factor to progress persuasion and comfortable settings between employees and association. Potential employees changed their minds about spreading their associates instead of strictly practicing information concealment [105]. Additionally, workers replaced the behavior of KH when older employee's imaginary administrative impartiality and dishonored inequality. Conclusions focused on eliminating undesirable associations with administrative fairness and relationship concealment; servant leadership performs a moderating role that weakens the direct connection when high servant leadership prevails. Because it easily exposes KH behavior, cynicism refers to the behavior of KH that makes prospective workforces redundant in an impartial setting. The results of this research are consistent with previous study showing that under servant leadership, employees morally focus on standards and ignore depraved repetition because servant leadership centers on societal duty [77]. Impulsively, servant leadership alters senior employees' views on job advancement, and they openly plan through their effort's assistants and partners as associates rather than competitors. Therefore, extreme servant leadership causes workplaces to be disturbed by a compassionate atmosphere and overlooks the behavior of KH through impartiality.

However, justice cannot always be indorsed in developing countries, and it was found in studies looking at KH behavior that such practices significantly affect employee performance. This needs to be considered when considering organizational design [56], and consequently, it misleads the staff into completing particular anxieties, which has a negative impact on the organizational context of emerging nations. Due to numerous assaults, long-standing agendas, and radical ideologies, this research is focused on Pakistan, making it impossible to shadow an accurate solution at this time. It has been discovered that KH can be stopped by properly implementing organizational design and justice, which will eventually improve performance.

### 5.2 Theoretical contributions and practical implications

This research has notable contributions and implications based on the present study constructs and their implemented mechanism. Based on the provoked contributions, this study used the TL theory of Burns [10], and the SE theory of Molm [18] to support its model. In light of this, determining the supplementary roles of organizational justice and 10.13039/100014294KH underwrite research supporting TL and SE theories. Few studies have been conducted that have focused on organizational justice and servant leadership as a curative force in the context of emerging nations, along with the behavioral phenomenon of KH. The study primarily contributed to the literature on servant leadership, which was used to moderate the relationship between organizational design and organizational justice as well as cynicism and KH. Additionally, organizational design can alter the independence of organizational justice by demonstrating standards, conviction, harm, and selflessness through appropriate tools created by workforce interpersonal behavior. Second, the current study significantly contributes to the literature by highlighting two intervening psychological constructs, organizational justice and KH, which will eventually boost employee performance.

Present study aims to minimize the common behavioral phenomenon of KH that can disrupt their social environment and eventually widen the gap between them. Currently, we promote systematic associations about the ineffectiveness of KH and use organizational justice and servant leadership as hopeful means of motivating workers to perform at their best on the job. The psychological, behavioral, and practically oriented constructs were arranged appropriately to measure performance, adding to the organizational context literature and making this research model unique. Finally, the results showed that organizational design had a significant effect on organizational justice, which could be initiated by influential servant leadership that positively influenced workers' productivity. However, cynicism has a beneficial influence on the behavior of KH, which can be the root of poor performance. In contrast to organizational design and justice, results showed that servant leadership significantly moderates the link between cynicism and KH.

Additionally, this study offers some noteworthy conclusions that aid organizational employees and practitioners in surviving in such a hostile environment. The current research initially suggested that assessing an employee's mental state is important because of human emotions and feelings. Diversity in this situation is a corrective measure to cover the irregularities that allowed the workers to improve. In order to encourage their workforce to compete through their arrangements that ultimately structure a supportive affiliation between workers, the organizational administration and older employees can create a culture by exemplifying organizational justice through servant leadership. Such supportive relationships between teams would encourage the practice of acquaintance sharing as opposed to KH and reassure a selfless strategy for their subordinate. The current study's practical implications align with previous studies that have shown that employees' knowledge-keeping behaviors keep them alive and that prejudicial motivations lead acquaintance keepers to steal from their peers [106].

The administrators and elderly employees must consider information concealment and modernized beating while adjusting their behavior. A changed arrest and a disadvantaged administration point to various conformations of keeping the information hidden. At that time, it is imperative to understand the origins of KH. In order to effectively control the behavior of withholding knowledge, administrators must also recognize the workforces with a high degree of servant leadership and a positive relationship with their dependents. According to servant leadership, different feedbacks are indicated by a worker's perception of managers and senior staff; consequently, different levels of implied fairness suppress the practice of KH. Additionally, executives must practice the impartial and ethical grounds to withstand the well-adjusted fund sharing while keeping the perspective of organizational justice in mind.

### 5.3 Limitations and future research directions

Even though this research refers to existing literature and has important theoretical and practical implications, it also has some limitations. Additionally, the limitations of each study paved the way for future research paths among potential scholars, organizations, and managerial authorities. First, Pakistan's manufacturing sector is covered by the present study variables. Similarly, in the data collection process, we used a cross-sectional technique that revealed the situation when the data was gathered. In the future, potential researchers will use a longitudinal data collection technique that more broadly generalizes the findings because respondents come from various attitudes that distress the contextual settings. Second, the current study sample was only 730, but it will be expanded to allow for more generalization. Thirdly, due to a lack of budgetary and time restraints, we only concentrate on Pakistani Manufacturing Organizations; however, in the future, a comparison with other developing or developed nations will be made to provide a clearer image on a global scale. Fourth, current study participants are trying to refrain from participating because of the nature of their jobs and their fear of disclosing their intellectual perceptions about KH and cynicism, which ultimately impact their future careers. Furthermore, in the future, potential researchers will incorporate a variety of knowledge-sharing constructs that freely provide unbiased responses to improve the study's generalizability. The use of additional performance-related attributes by potential investigators due to the development of technology in recent years has led to a shift towards knowledge sharing over 10.13039/100014294KH, with the vital support of justice-related facets.

## Ethics statement

No animal studies are presented in this manuscript. “This study involving human participants was reviewed and approved by The TIMES Institute, Office of Research, Innovation and Commercialization (ORIC), Multan, Pakistan Ref No (TI/ORIC/2023/73). The participants provided their written informed consent to participate in this study.”

## Data availability

Data will be made available on request.

## CRediT authorship contribution statement

**Abdul Rauf:** Writing – original draft, Methodology, Conceptualization. **Hamid Mahmood:** Writing – review & editing, Validation, Resources. **Rana Tahir Naveed:** Validation, Software, Resources, Data curation. **Yuen Yee Yen:** Writing – original draft, Software, Methodology, Conceptualization.

## Declaration of competing interest

The authors declare that they have no known competing financial interests or personal relationships that could have appeared to influence the work reported in this paper.
