# Supplementary material for: Modeling cynicism and organizational design on job performance: Mediation and moderation mechanism
Source: Heliyon. 2024 May 29;10(11):e32069. doi: 10.1016/j.heliyon.2024.e32069 (PMC11176861; doi:10.1016/j.heliyon.2024.e32069)
Supplement: Multimedia component 1 [file mmc1.docx]

**Modeling of cynicism and organizational design on job performance: Mediation and moderation mechanism**

The aim of the study is to investigate how cynicism affects job performance through organizational design. It also explores factors that may strengthen or weaken this relationship.

Dear respondent, kindly fill up this information and return. Any information obtain for this purpose will be kept strictly confidential and will only be used for academic purpose. Your cooperation will be highly appreciated in this regard.

What is your Gender?

1. Male B) Female

Which age group do you belong?

1. 18-25 Years D) 42-49
2. 26-33 E) 50 or above
3. 34-41

What is your Qualification?

1. Intermediate C) Masters

B) Bachelor D) Mphil

What is your Income?

1. 15,000-24000 PKR C) 34001-44000 PKR
2. 24,001-34000 PKR D) 44,001 or above

What is your Experience?

1. 1 - 3 Years C) 7 - 9 Years
2. 4 - 6 Years D) 10 and above

**A**. You are required to indicate your level of personal for Organizational Justice on a Seven-point Likert scale from 1 to 7.

| **Organizational Justice** | | **1** | **2** | **3** | **4** | **5** | **6** | **7** |
| --- | --- | --- | --- | --- | --- | --- | --- | --- |
| **1** | I consider my work load to be quite fair. |  |  |  |  |  |  |  |
| **2** | I think that my level of pay is fair. |  |  |  |  |  |  |  |
| **3** | Job decisions are made by the general manager in an unbiased manner. |  |  |  |  |  |  |  |
| **4** | All job decisions are applied consistently across all affected employees. |  |  |  |  |  |  |  |
| **5** | When decisions are made about my job, the general manager treats me with kindness and consideration. |  |  |  |  |  |  |  |
| **6** | When decisions are made about my job, the general manager shows concern for my rights as an employee. |  |  |  |  |  |  |  |

**B**. You are required to indicate your level of personal for Knowledge Hiding on a Seven-point Likert scale from 1 to 7.

| **Knowledge Hiding** | | **1** | **2** | **3** | **4** | **5** | **6** | **7** |
| --- | --- | --- | --- | --- | --- | --- | --- | --- |
| **1** | Are you really never intended to help others? |  |  |  |  |  |  |  |
| **2** | Promise that you would help but trying to delayed as possible. |  |  |  |  |  |  |  |
| **3** | Offered irrelevant information instead of needed one. |  |  |  |  |  |  |  |
| **4** | Are you pretending that you have no information? |  |  |  |  |  |  |  |
| **5** | Even you did but trying to convince that you did not know. |  |  |  |  |  |  |  |
| **6** | Even you know but pretend that what you talking about. |  |  |  |  |  |  |  |

**C.** You are required to indicate your level of personal for Organizational Design on a Seven-point Likert scale from 1 to 7.

| **Organizational Design** | | **1** | **2** | **3** | **4** | **5** | **6** | **7** |
| --- | --- | --- | --- | --- | --- | --- | --- | --- |
| **1** | Job design with participation in decision-making. |  |  |  |  |  |  |  |
| **2** | Job design with high levels of autonomy. |  |  |  |  |  |  |  |
| **3** | Layout design with architectural privacy in the workspace. |  |  |  |  |  |  |  |
| **4** | Territorial design inducing need of knowledge protection. |  |  |  |  |  |  |  |
| **5** | IT infrastructure to externally acquire information. |  |  |  |  |  |  |  |
| **6** | Knowledge sharing embedded in everyday work practices. |  |  |  |  |  |  |  |
| **7** | Layout design with satisfying physical proximity of the workstations. |  |  |  |  |  |  |  |
| **8** | Layout design with equal privacy setting of the workstations. |  |  |  |  |  |  |  |
| **9** | Layout design with equal space for the workstations. |  |  |  |  |  |  |  |

**D.** You are required to indicate your level of personal for Cynicism on a Seven-point Likert scale from 1 to 7.

| **Cynicism** | | **1** | **2** | **3** | **4** | **5** | **6** | **7** |
| --- | --- | --- | --- | --- | --- | --- | --- | --- |
| **1** | I have become less enthusiastic about my work. |  |  |  |  |  |  |  |
| **2** | I have become more cynical about whether my work contributes anything. |  |  |  |  |  |  |  |
| **3** | I doubt the significance of my work. |  |  |  |  |  |  |  |
| **4** | I have become less interested in my work. |  |  |  |  |  |  |  |
| **5** | I lost my concentration and was bothered by other matters. |  |  |  |  |  |  |  |

**E.** You are required to indicate your level of personal for Servant Leadership on a Seven-point Likert scale from 1 to 7.

| **Servant Leadership** | | **1** | **2** | **3** | **4** | **5** | **6** | **7** |
| --- | --- | --- | --- | --- | --- | --- | --- | --- |
| 1 | My department manager creates a sense of community among department employees. |  |  |  |  |  |  |  |
| **2** | My department manager’s decisions are influenced by department employees’ input. |  |  |  |  |  |  |  |
| **3** | My department manager makes the personal development of department employees a priority. |  |  |  |  |  |  |  |
| **4** | My department manager does what she or he promises to do. |  |  |  |  |  |  |  |
| **5** | My department manager works hard at finding ways to help others be the best they can be. |  |  |  |  |  |  |  |

**F.** You are required to indicate your level of personal for Job Performance on a Seven-point Likert scale from 1 to 7.

| **Job Performance** | | **1** | **2** | **3** | **4** | **5** | **6** | **7** |
| --- | --- | --- | --- | --- | --- | --- | --- | --- |
| 1 | I fulfilled my job responsibilities. |  |  |  |  |  |  |  |
| **2** | I met performance standards and expectations of the job. |  |  |  |  |  |  |  |
| **3** | My performance level satisfied my manager. |  |  |  |  |  |  |  |
| **4** | I was effective in my job. |  |  |  |  |  |  |  |
| **5** | My performance was still good as the time before. |  |  |  |  |  |  |  |

***Thank you…..***
